# Incidence, pathogens and resistance patterns of nosocomial infections at a rural hospital in Gabon

**DOI:** 10.1186/1471-2334-14-124

**Published:** 2014-03-04

**Authors:** Micha Scherbaum, Katrin Kösters, Raymund Egid Mürbeth, Ulysse Ateba Ngoa, Peter Gottfried Kremsner, Bertrand Lell, Abraham Alabi

**Affiliations:** 1 Centre de Recherches Medicales de Lambarene, Albert Schweitzer Hospital, PB 118 Lambaréné, Gabon; 2 Department for Tropical Medicine, Eberhard-Karls-University Tübingen, Wilhelmstraße 27, D-72074 Tübingen, Germany; 3 Department of Internal Medicine II, Helios Clinic Krefeld, Lutherplatz 40, 47805 Krefeld, Germany; 4 Third Medical Clinic, Augsburg Hospital, Stenglingstr 2, 86156 Augsburg, Germany

**Keywords:** Nosocomial infections, Antibiotic resistance, Gabon

## Abstract

**Background:**

Nosocomial infections pose substantial risk to patients receiving care in hospitals. In Africa, this problem is aggravated by inadequate infection control due to poor hygiene, resource and structural constraints, deficient surveillance data and lack of awareness regarding nosocomial infections. We carried out this study to determine the incidence and spectrum of nosocomial infections, pathogens and antibiotic resistance patterns in a tertiary regional hospital in Lambaréné, Gabon.

**Methods:**

This prospective case study was carried out over a period of six months at the Albert Schweitzer Hospital, Lambaréné, Gabon. All patients admitted to the departments of surgery, gynecology/obstetrics and internal medicine were screened daily for signs and symptoms of hospital-acquired infections.

**Results:**

A total of 2925 patients were screened out of which 46 nosocomial infections (1.6%) were diagnosed. These comprised 20 (44%) surgical-site infections, 12 (26%) urinary-tract infections, 9 (20%) bacteraemias and 5 (11%) other infections. High rates of nosocomial infections were found after hysterectomies (12%) and Caesarean sections (6%). Most frequent pathogens were *Staphylococcus aureus* and *Escherichia coli*. Eight (40%) of 20 identified *E. coli* and Klebsiella spp. strains were ESBL-producing organisms.

**Conclusion:**

The cumulative incidence of nosocomial infections in this study was low; however, the high rates of surgical site infections and multi-resistant pathogens necessitate urgent comprehensive interventions of infection control.

## Background

Health-care-associated infection (HAI), otherwise known as nosocomial infection, is a major global safety concern for both patients and health-care professionals [1-3]. Excess morbidity, mortality and costs of illnesses caused by nosocomial infections have been demonstrated by several studies [1-7]. In the developed countries, it has been reported to affect from 5% to 15% of hospitalized patients in regular wards and as many as 50% or more of patients in intensive care units (ICUs), while in developing countries the magnitude of the problem remains largely underestimated [3,8]. Only few studies have focused on nosocomial infections in developing countries, especially in Sub-Saharan Africa [9,10]. As shown in a recent meta-analysis of 220 studies from developing countries (of which only 14 studies were from Africa) high rates of nosocomial infections with an incidence of 7.4 infections per 100 patients were found [10].

Urinary-tract infections represent the most frequent form of nosocomial infections in industrialized nations, while in developing countries invasive medical procedures play a major role [1,11,12]. Surgical interventions are one of the biggest sources of nosocomial infections with an incidence ranging from 1.2% to 23.6% of all surgical interventions and *Staphylococcus aureus* (20%), *Escherichia coli* (18%) and other Enterobacteria constituting the most frequent causative pathogens in developing countries [10,13-15]. Apart from patient-related factors (e.g. co-infection of other local sites, malnutrition and/or immune-deficiency), the quality of equipment, financial resources as well as the competence of the surgical team seem to be important determinants [13].

In developing countries a growing proportion of nosocomial infections can be assigned to methicillin-resistant *S. aureus* (MRSA) and multi-resistant Gram-negative bacteria [12,14]. In a recent study in Tanzania [15], surgical site infection (SSI) was detected in 65 (26.0%) patients, of whom 56 (86.2%) and 9 (13.8%) had superficial and deep SSI respectively. Among 65 patients with clinical SSI, 56(86.2%) had positive aerobic culture. *S. aureus* was the predominant organism 16/56 (28.6%); of which 3/16 (18.8%) were MRSA. This was followed by *Escherichia coli* 14/56 (25%) and *Klebsiella pneumoniae* 10/56 (17.9%). Among the *E. coli* and *K. pneumoniae* isolates 9(64.3%) and 8(80%) were ESBL producers respectively. In Niger, a study reported up to 31% ESBL colonization rates on hospital admission [16].

The rapid spread of antibiotic-resistance poses an immense threat to health systems of these countries where adequate antibiotic treatment of infectious diseases is often hampered by financial constraints [17-19]. For Gabon and most of Central Africa, data on nosocomial infections are lacking; hence this study was carried out to estimate the incidence of hospital-acquired infections and characterise the pathogens responsible at a rural hospital in Central Africa as a first step to reduce nosocomial infections.

## Methods

### Study area

Lambaréné has approximately 24000 inhabitants and lies on the Ogooué River, in a region predominantly covered by dense tropical rain forest in Central Africa. The Albert Schweitzer Hospital, a 150 bed health facility, has a catchment population of about 50000 persons and provides surgical, internal medical, obstetrics/gynecological and pediatric services.

### Study procedures

We undertook a prospective surveillance study in order to determine the incidence of nosocomial infections at the Albert Schweitzer Hospital. The study was carried out in the departments of aseptic surgery (clean and clean contaminated surgery), septic surgery (contaminated surgery), gynecology/obstetrics and internal medicine with a total of 114 beds. The study lasted six months from January to July 2009. On daily rounds all patients were screened daily for signs and symptoms of nosocomial infections. Patients with a new occurrence of fever (> 38°C) at least 48 hours after admission or a repeated episode of fever after a non-febrile interval of 72 hours, signs of infection at the site of surgical operations, or a reopening of the operation site due to infection qualified for further evaluation. Further evaluation consisted of a clinical examination, and personal data, reason for hospitalization, operations, current signs and symptoms, and inserted venous or urinary catheters were recorded. Days from admission to occurrence of fever or other signs and symptoms of nosocomial infections were noted.

Based on clinical signs and symptoms, specific laboratory tests were performed. For every patient with fever (> 38°C), blood and urine cultures as well as parasitological examination of thick blood smear for exclusion of malaria were carried out. In case of a suspected surgical site infection a wound swab was obtained and processed in the microbiology laboratory. Specific symptoms like diarrhoea or abnormal breathing sounds led to stool examinations or chest x-ray, respectively. To ensure that an additional nosocomial infection of the same patient had originated independently from a previous one, the patient had to be symptom free for at least 72 hours or the infection had to affect a different localisation or grow a different pathogen. Classification of cases occurred according to the definitions of nosocomial infections of the Robert Koch Institute (RKI) in Berlin which were based on the current CDC guidelines [20]. These criteria give an epidemiological definition of nosocomial infections to be able to compare surveillance data from different studies. The time for occurrence of nosocomial infection was noted and analysed; and patients who had no nosocomial infection at the time of initial screening were followed up daily for symptoms of nosocomial infections until they were discharged. Patients with nosocomial infections were also followed up until discharge.

### Laboratory procedures

Microbiological specimens were processed according to good laboratory practice and standard methods for identification. Briefly, blood cultures were collected in Bactec blood culture bottles (BD Blood Culture System, Becton, Dickinson and Company) and incubated at 37°C in Bactec 9050. Positive bottles were subcultured on blood, chocolate and MacConkey agar. Swabs and urine were plated on blood and MacConkey agar. Bacteria were identified by tube coagulase and latex tests for staphylococci, latex test for streptococci, pyrase testing for enterococci and API-20E and API-20NE (Biomeriux) for enterobacteriaceae and non-fermenting Gram-negative rods respectively. Susceptibility to antibiotics was tested using the microdilution method according to CLSI guidelines [21] Inducible clindamycin resistance is not routinely tested in our laboratory and therefore not reported. Antibiotics tested were chosen according to local availability and included penicillin, oxacillin, ampicillin, amoxicillin/clavulanic acid, ceftriaxon, gentamicin, ciprofloxacin, co-trimoxazole, clindamycin, chloramphenicol and erythromycin. Extended Spectrum β-Lactamase (ESBL) production was confirmed by disc diffusion test. The production of extended-spectrum beta-lactamase was confirmed in all ceftriaxone resistant Enterobacteriaceae using the double-disks method according to the manufacturer’s instruction (Mast discs, Mast diagnostics, Merseyside, UK). This test applies discs of three different beta-lactam antibiotics (ceftazidim 30 μg, cefotaxime 30 μg, cefpodoxime 10 μg) with and without clavulanic acid. Methicillin-resistance was confirmed for all cefoxitin-resistant *Staphylococcus aureus* using a PBP2a-agglutination test (PBP2’ Test Kit, Oxoid, Japan).

### Data analysis

Data collected from patients with signs and symptoms of nosocomial infections (age, sex, length of hospital stay, type of nosocomial infection, days to first clinical sign or symptom of nosocomial infection, type of operation if applicable) were entered in a Microsoft® Access 2002 data bank. Data on all hospitalised patients was available for either individual patients (sex, age) or aggregated over a month (mean duration of stay). For statisical analysis, IBM SPSS® Statistics (version 19) was used. The continuous variables were expressed as median (25-75% quantiles) and compared between groups using the Mann–Whitney U test. Categorical variables were expressed in percentage (proportion). Statistical analysis was supervised by a statistician at the Institute of Medical Biometrics and Medical Informatics for Biometrics, Albert’s Ludwig University of Freiburg, Germany.

### Ethics

The study was approved by the Regional Ethical Committee, “Comité d’Ethique Régional Independent de Lambaréné (CERIL)”. Written informed consent was obtained from all patients’ prior inclusion into the study.

## Results

During the six months period of this study, 2925 patients were admitted to the participating wards of the Albert Schweitzer Hospital. The mean duration of stay was 4.4 days resulting in 12870 hospital days.

The median age was 30 (22–42) years, 65% of all patients were female and 35% were male. In total, 84 patients met our criteria for further evaluation for suspected nosocomial infections and 46 nosocomial infections were confirmed in 36 patients out of 2925 inpatients. A single nosocomial infection was diagnosed in 30 patients, two infections were found in three patients, three infections in two patients, and four infections in one patient. Forty eight patients who were further evaluated did not have a nosocomial infection as defined by the epidemiological criteria of the RKI. Forty (87%) episodes of nosocomial infections were confirmed by microbiological examinations, while the remaining six infections were diagnosed on clinical grounds only. During the study four (11%) of the patients with nosocomial infections died.

The cumulative incidence of nosocomial infections was 1.6 infections per 100 patients in six months (95% CI 1.1 - 2.0) or 3.6 infections per 1000 in-patient days (95% CI 2.6 – 4.6). Details about the incidence of nosocomial infections at the respective departments are shown in Table 1. With a total of 20 cases (44%) surgical-site infections were the most frequent type of hospital-acquired infection, followed by twelve (26%) urinary-tract infections (of which 4 were catheter related infections), nine (20%) bloodstream infections and five (11%) other infections (e.g. diarrhoea, phlebitis, decubitus ulcer infection).

**Table 1:** Incidence of nosocomial infections at the hospital departments

**Department**	**No. nosocomial infections/no. patients**	**Cumulative incidence [%, 95% CI]**	**Incidence density**	**Surgical-site infection**		**Urinary-tract infection**		**Bloodstream infection**	
			**[per 1000 in-patient days, 95% CI]**	**Incidence [%, 95% CI]**	**Incidence density [per 1000 in-patient days, 95% CI]**	**Incidence [%, 95% CI]**	**Incidence density [per 1000 in-patient days, 95% CI]**	**Incidence [%, 95% CI]**	**Incidence density [per 1000 in-patient days, 95% CI]**
Surgery	31/1022	3.0% (2.0 – 4.1)	5.1 (3.3 - 6.9)	1.4 (0.7 - 2.1)	2.3 (1.1 – 3.5)	1.1 (0.5 - 1.7)	1.8 (0.8 – 2.8)	0.2 (0.0 - 0.5)	0.3 (0 – 0.7)
Aseptic^1)^ surgery	20/689	2.9% (1.6 – 4.2)	8.6 (4.9 - 12.3)	1.9 (0.8 - 2.9)	5.6 (2.6 – 8.5)	0.7 (0.1 - 1.4)	2.1 (0.2 – 4.1)	0.1 (0–0.4)	0.4 (0 – 1.2)
Septic^2)^ surgery	11/333	3.3% (1.4 - 5.2)	2.9 (1.2 - 4.7)	0.3 (0–0.9)	0.3 (0 – 0.8)	1.8 (0.4 - 3.2)	1.6 (0.2 – 3.0)	0.3 (0–1.9)	0.3 (0 – 0.9)
Gynaecology	12/1047	1.1% (0.5 - 1.8)	4.0 (1.8 - 6.1)	0.6 (0.1 - 1.0)	2.0 (0.4 – 3.6)	0.1 (0.0 - 0.3)	0.3 (0 – 0.9)	0.5 (0.1 - 0.9)	1.7 (0.3 – 3.0)
Internal medicine	3/856	0.4% (0.0 - 0.7)	0.8 (0–1.8)	0 (-)	0 (-)	0 (-)	0 (-)	0.2 (0.0 - 0.5)	0.5 (0 – 1.3)
All departments	46/2925	1.6% (1.1 - 2.0)	3.6 (2.6 - 4.6)	0.7 (0.4 - 1.0)	1.6 (1.0 – 2.1)	0.4 (0.2 - 0.6)	0.9 (0.4 – 1.5)	0.3 (0.1 - 0.5)	0.7 (0.3 – 1.1)

^1)^Clean and clean-contaminated surgery.

^2)^Contaminated and dirty-infected surgery.

During the study period, a total of 1059 surgical operations on 992 patients were performed. The finding of 20 surgical-site infections corresponded to an overall infection rate of 1.9% (95% CI 1.1 - 2.7%) per operation. In Table 2, the number and incidence of nosocomial infections per type of surgical operation are shown for the most frequently performed aseptic operations. Abdominal hysterectomies had the highest incidence of nosocomial infections with 11.5% (95% CI 0.0 - 23.8).

**Table 2:** Incidence of surgical-site infections per operation type

**Type of surgical operation**	**No. surgical-site infection/no. operations**	**Incidence [%, 95%CI]**
Abdominal hysterectomy	3/26	11.5 (0.0 - 23.8)
Caesarean section	5/80	6.3 (0.9 - 11.6)
Appendectomy	1/17	5.9 (0.0 - 17.1)
Myomectomy	1/19	5.3 (0.0 - 15.3)
Salpingectomy (ectopic pregnancy)	1/22	4.5 (0.0 - 13.2)
Herniotomy	2/124	1.6 (0.0 - 3.8)

Patients with nosocomial infections had a median age of 41 (33–69)years, which is significantly higher than the age of patients who did not acquire infections during hospitalisation 30 (22–41) years, p <0.001). Regarding the incidence of nosocomial infections, no significant difference in sex was found (p = 0.08). The duration of in-patient care of patients with nosocomial infections ranged from four to 108 days with a median of 22 (13–34) days. The first clinical signs of nosocomial infection appeared after a median of 6 (4–10) days of patient care. Approximately half (24/46) of all nosocomial infections developed under antibiotic treatment.

In total, 68 blood cultures, 67 urine cultures and 52 wound swabs were sent for microbiological evaluation. As shown in Table 3, 36 Gram-positive and 34 Gram-negative bacteria were identified in 40 specimens. The most frequent pathogen was *S. aureus*, in most cases originating from surgical-site infections, followed by *E. coli* as a cause of nosocomial urinary-tract infections and Enterococcus spp. from surgical-site infections. *K. pneumoniae* was identified from urinary-tract and bloodstream infections, while anaerobes were exclusively found in specimen from surgical sites.

**Table 3:** Pathogens with antibiotic sensitivity

**Pathogen**	**No. of pathogens**	**No. detected in surgical-site infections**	**No. detected in urinary-tract infections**	**No. detected in bloodstream infections**	**Antibiotics (No. of sensitive pathogen/no. of tested pathogens)**							
					**Penicillin**	**Cloxacillin**	**Ampicillin**	**Amoxicillin + Clavulanic acid**	**Ceftriaxone**	**Gentamicin**	**Ciprofloxacin**	**Cotrimoxazole**
*Staphylococcus aureus*	15 (21.4%)	9	0	5	*0/14	14/14						
*Escherichia coli*	14 (20.0%)	3	8	1	-	-	1/14	6/14	9/14	9/14	9/14	3/12*
Enterococcus	9 (12.9%)	5	4	0	0/9	-	9/9	-	-	-	-	-
*Klebsiella pneumoniae*	6 (8.6%)	0	3	3	-	-	0/6	2/6	3/6	3/6	3/6	1/5*
Anaerobes	6 (8.6%)	6	0	0	-	-	-	-	-	-	-	-
Acinetobacter species	4 (5.7%)	1	3	0	-	-	0/4	1/4	0/4	2/4	3/4	1/4
*Streptococcus pyogenes*	3 (4.3%)	2	0	1	3/3	-	3/3	-	-	-	-	-
Other bacteria^#^	13 (18.6%)	11	2	0	-	-	-	-	-	-	-	-

*Antibiotic resistance testing was not performed for some pathogens.

^#^Bacillus spp, Prevotella spp., *Citrobacter koseri*, Corynebacterium spp, *Proteus mirabilis*, *Pseudomonas aeruginosa*, Serratia spp, coagulase-negative staphylococci.

The antibiotic susceptibility profiles of the nosocomial pathogens are detailed in Table 3. A total of eight multi-resistant strains of Gram-negative bacteria with ESBL-production were detected, comprising five *E. coli* and three *K. pneumoniae* strains. Four of the ESBL-producing bacteria were resistant to the whole spectrum of antibiotics available at the Albert Schweitzer hospital. Six ESBL-positive bacteria caused nosocomial infections while the patients were on continued perioperative prophylactic antibiotic treatment with an aminopenicillin w/o ß-lactamase inhibitor or ceftriaxon.

## Discussion and conclusions

In many developing countries particularly in sub-Saharan Africa, data about the incidence of hospital-acquired infections at rural hospitals are lacking, as modern microbiological laboratories are generally restricted to hospitals in major cities. Between January and July 2009 we performed daily ward rounds in the departments of surgery, internal medicine and obstetrics/gynecology to document all nosocomial infections as defined by the CDC criteria. The overall rate of nosocomial infections at the Albert Schweitzer hospital excluding the pediatric department was 46 cases in 12870 hospital days corresponding to an incidence density of 3.6 per 1000 in-patient days. With nearly 44% of all cases, surgical-site infections were the most frequently diagnosed nosocomial infections, followed by urinary-tract infections (26%), bloodstream infections (20%) and other infections (11%). This is in agreement with findings in other studies from developing countries where surgical-site infections made up the majority of hospital-acquired infections [1,9,14,15]. The missing evidence of nosocomial respiratory tract infections as defined by the CDC criteria during the study period could be explained by the absence of an intensive-care unit with invasive ventilation procedures [22,23] and the restricted availability of the X-ray machine.

We found a very low overall nosocomial infection incidence rate as compared with the average incidence of nosocomial infections in developing countries of 7.4% [8,14,15]. Possible reasons for this could be the small size of the hospital (114 beds) and the on-average short duration of in-patient stay (mean 4.4 days). The absence of an intensive care unit, the limited spectrum of operations and the fact that certain invasive procedures like insertion of central venous catheters were not performed could also have contributed to a comparatively low incidence of hospital-acquired infections at the Albert Schweitzer hospital. On the other hand it could also be due to a high level of awareness of the need for aseptic procedures among doctors and nurses at our hospital.

The finding of 20 surgical-site infections corresponded to an infection rate of 1.9 (95% CI 1.1 - 2.7%) per 100 surgical procedures. This was also lower than the pooled cumulative incidence of 5.6% reported from other developing countries [10] or 26% reported from Tanzania [15]. But the infection rates for specific surgical operations such as caesarean sections (6.3%) and hysterectomies (11.5%) clearly were above the rates reported from American surveillance studies (1.5% - 3.8% for caesarean sections and 1.1% - 4.1% for hysterectomies) [24]. The low overall rate was probably due to the fact that most operations performed at the Albert Schweitzer hospital were low risk procedures and surgical procedures with a high risk of nosocomial infection as found in Tanzania (open prostatectomy, mastectomy, spina bifida repair) [15] were not done. This emphasizes the fact that for meaningful comparisons of nosocomial infection risks, hospitals with similar patient characteristics and invasive procedures have to be compared.

While the most frequent pathogens of nosocomial infections at the Albert Schweitzer hospital were similar to data from other countries, the resistance patterns of certain strains of bacteria were alarming. The finding that *E. coli*, as the second-most isolated pathogen in Lambaréné, was sensitive to ampicillin in only one of 14 cases (i.e. 7%, 95% CI 0 – 21%) and that sensitivity to broad spectrum antibiotics such as ceftriaxone, ciprofloxacin and gentamicin was found in only nine out of 14 cases (95% CI 39 - 89%) is of particular concern. Similarly worrisome is the reduced sensitivity of *K. pneumoniae* strains, of which only half were sensitive to ceftriaxone, ciprofloxacin and gentamicin. These alarming resistance rates may reflect the high use of antibiotics either in hospital or as outpatients in a country where antibiotics are freely available over the counter.

One of our most striking findings was the fact that five of 14 E. coli strains (36%) and three of six *K. pneumoniae* strains (50%) were ESBL-producers. Similar resistance rates have been described in screening isolates and clinical isolates in Tanzania (15), Ghana [25], Cameroon [26] and Sudan [27], Four of the ESBL-producing bacteria were resistant to the whole spectrum of antibiotics available at the Albert Schweitzer hospital. While colonisation with MRSA was identified in two patients, MRSA causing nosocomial infections was not found. This observed high rate of antibiotic resistance among our isolates is in agreement with a review of published literature on bacteria resistance in Central Africa (1955–2008) showing that the Central African region shares the worldwide trend of increasing antimicrobial resistance [28,29]. There is an urgent need of sound surveillance based on competent and affordable microbiology to provide clear and fairly frequent data on antimicrobial resistance.

More than half of all nosocomial bacterial infections acquired in the Albert Schweitzer hospital were diagnosed in patients under antibiotic medication. Similar observations have been reported from other African countries, like Ethiopia, where 72% of nosocomial infections developed under antibiotic prophylaxis [30]. In the present study six of eight cases of nosocomial infections with ESBL-producing bacteria occurred while the patients were on prophylactic or therapeutic antibiotic therapy already. These findings support the view that inappropriate use of anti-infectious medications including routine antibiotic prophylaxis of long duration is a major factor favouring infection with multi-resistant pathogens [16-19,25-28].

With a mean stay of 27 (range: 4–108) days, patients with nosocomial infections stayed in hospital for 23 days longer than the average patient. This may partly be explained by the hypothesis that patients developing nosocomial infections have more co-morbidities which lead to longer inpatient care per se and therefore exposes them to the risk of hospital-acquired infections for a longer time period. This hypothesis is supported by the fact that initial clinical manifestations of nosocomial infections were observed on median day 8 of hospitalization which is twice the average duration of stay of all patients. At the same time nosocomial infections also have been reported to lead to prolonged hospital stay [31].

There are several limitations to this study. Hospital -acquired infections that arose after discharge were not detected due to lack of follow-up. Because a temperature >38°C was the main inclusion criterion except for patients with a surgical site infection we might have underestimated the rate of nosocomial infections in patients receiving antipyretics.

The results of this study revealed that nosocomial infections represent a substantial threat for patients at the Albert Schweitzer hospital. Although the overall incidence rate of nosocomial infections was lower than in hospitals from other developing countries we found several areas for improvement. At the Albert Schweitzer hospital, a particular high risk of nosocomial infections was found after gynecological operations. Thus, interventions to decrease hospital-acquired infections by e.g. infection control or appropriate perioperative prophylaxis should start here. This study also showed that hospital wide nosocomial infection rates have to be interpreted carefully because they depend on the services offered at the respective hospital and on patient characteristics.

Many of the identified pathogens, particularly the Gram-negative bacteria have developed resistance to commonly prescribed antibiotics. Moreover, against ESBL-strains identified in this study no effective antibiotics were available at the Albert Schweitzer hospital. This means antibiotics to deal with infections with multiresistant bacteria have to be made available and in the long run interventions to reduce the rate of multiresistant pathogens have to be taken. These could, for example, consist of introducing antibiotic stewardship measures which includes appropriate use of antibiotic medications in the hospital setting [28]. These strategies can only be accomplished by quality management involving different disciplines, and capacity building of the personnel. We also showed that at small rural hospitals detailed surveillance of nosocomial infections is mandatory to be able to reduce hospital-acquired infections and improve patient outcome.

## Competing interests

All authors declare that they have no competing interests.

## Authors’ contributions

MS made contributions to the study design and lead data acquisition as well as analysis and interpretation of data. He also drafted the manuscript. KK designed the study and made substantial contributions to analysis and interpretation of data. She was involved in drafting and in critical revision of the manuscript. REM designed the study and contributed to data acquisition as well as revision of the manuscript. UAN took part in data acquisition and revision of the manuscript. PGK took part in study design as well as critical revision of the manuscript. BL contributed study design, analysis and interpretation of the data and revision of the manuscript. AA took part in critical revision of the manuscript. All authors read and approved the final manuscript.

## Pre-publication history

The pre-publication history for this paper can be accessed here:

http://www.biomedcentral.com/1471-2334/14/124/prepub
